# DECODE VRL Report No. 1: Lymphocyte Survival After Diagnostic Vitrectomy – Does Size Matter?

**DOI:** 10.1167/tvst.15.1.31

**Published:** 2026-01-27

**Authors:** Martin Kowalski, Vinodh Kakkassery, David Adrian Merle, Florian Heubach, Alexandros Athanasiou, Spyridon Dimopoulos, Stefanie Paigin, Falko Fend, Karl-Ulrich Bartz-Schmidt, Friederike Charlotte Kortuem

**Affiliations:** 1 University Eye Hospital, Centre for Ophthalmology, University Hospital Tübingen, Tübingen, Germany; 2 Department of Ophthalmology, Klinikum Chemnitz, Germany; 3 Department of Pediatric Hematology and Oncology, University Children’s Hospital Tübingen, Tübingen, Germany; 4 Institute of Pathology and Neuropathology, University Hospital Tübingen and Eberhard Karls University Tübingen, Tübingen, Germany

**Keywords:** vitreoretinal lymphoma, vitreous biopsy, lymphocytes, fluorescence activated cell sorting (FACS), lymphoma cells

## Abstract

**Purpose:**

The purpose of this study was to assess whether vitrectomy size and cutting rate have an impact on cell survival of healthy lymphocytes and lymphoma cells during diagnostic vitrectomy in an in vitro model.

**Methods:**

In the first experiment, healthy lymphocytes were isolated from blood samples of one healthy individual. In the second experiment, cultivated *MYD88*-positive lymphoma cells derived from a commercially available cell line were used. Suspensions with defined cell concentrations (1 × 10^3^ cells/mL, 1 × 10^4^ cells/mL, and 1 × 10^6^ cells/mL) were subjected to vitrectomy under controlled conditions, using aspiration only or varying cutting rates of 1500/minutes and 5000/minutes with vitrectomy sizes of 20G, 23G, 25G, and 27G. Three technical replicates were performed for each condition. Cell integrity in post-vitrectomy samples was assessed via fluorescence activated cell sorting (FACS). To verify statistically significant differences between groups, the Kruskal-Wallis test was used.

**Results:**

No correlation was observed between lymphocyte survival and either gauge size or lower cutting rates in healthy lymphocytes. However, a significant correlation between vitrectomy size and cell survival was observed for lymphoma cells (Kendall's tau τ = −0.253, *P* = 0.00048, Kruskal-Wallis χ² = 13.337, *P* = 0.00396).

**Conclusions:**

Our study found that larger vitrectomy lumen diameters were associated with higher lymphoma cell survival rates, suggesting the use of 23G and 25G instruments in clinical practice for suspected cases.

**Translational Relevance:**

This clinical research should provide a guideline for the investigation of suspected lymphoma cases.

## Introduction

Vitreoretinal lymphoma (VRL) is a rare, aggressive subtype of diffuse large B-cell lymphoma (DLBCL) that primarily affects ocular structures such as the vitreous, retina, and subretinal space. It is strongly associated with the central nervous system (CNS). VRL manifests in two distinct forms. The first is primary VRL (PVRL), which originates in ocular tissue (retina/vitreous), and is biologically linked to primary CNS lymphoma (PCNSL). Second, a secondary VRL, occurs as metastatic spread from systemic lymphomas (e.g. testicular DLBCL).^1^ With 0.047 cases per 100,000 people per years,^2^ it is classified as an orphan disease (ORPHA:279904), but is nevertheless responsible for 4% to 6% of all brain tumors and less than 1% of all non-Hodgkin lymphomas.^1^^–^^4^

Presenting often as an unspecific uveitis masquerade syndrome with blurred vision, floaters, and intraocular infiltration, the rate of misdiagnosis up until today remains high.^5^ A diagnostic pars plana vitrectomy (PPV) with removal of a vitreous sample for a subsequent pathological analysis is currently often regarded as the standard procedure in diagnosis.^6^ Literature reports the sensitivity of vitrectomy to be 77% and the specificity to be 73%.^7^

With the advent of liquid biopsy, it is worth questioning if invasive surgery is still needed.^8^^,^^9^ Whereas both PPV and vitreous tap remain valuable diagnostic tools, each offers distinct advantages.^10^^,^^11^ PPV provides superior sample quality and quantity, yielding undiluted specimens with significantly higher cellularity than other methods.^4^^,^^12^ This increased cellularity enhances diagnostic accuracy, potentially reducing false negative results that occur in 30% to 45% of non-PPV cytology analyses.^13^^,^^14^ PPV also enables comprehensive diagnostic capabilities, including detailed cytological examination.^4^^,^^12^ Furthermore, PPV offers therapeutic benefits by clearing vitreous debris and improving vision.^4^^,^^15^ Conversely, vitreous tap offers a less invasive alternative, which may be preferable in certain clinical scenarios.^9^^–^^11^ The choice between these methods depends on factors such as patient condition, clinician expertise, and institutional protocols. The optimal approach for VRL diagnosis may involve a tailored combination of these methods, based on individual patient needs and clinical circumstances.

Even with a good vitreous sample, diagnosis can be difficult for the ophthalmo-pathologist due to low biopsy volume, low cell counts, and the fragility of vitreous lymphoma cells.^14^ Data show that about one-third of negative cases are falsely negative.^6^^,^^14^ This diagnostic gap often requires multiple diagnostic procedures, delaying the histopathological confirmation of diagnosis.^16^^,^^17^ Not infrequently even retinal biopsies within the setting of a vitrectomy become necessary. This shows the need for a standardization of a vitreous biopsy in cases suspicious for malignancy as delaying the diagnosis might have vision or even life-threatening consequences.^18^

The DECODE-VRL study (NCT05841914) aims to address this diagnostic gap in suspected vitreoretinal lymphoma cases and enhance diagnostic accuracy and patient outcomes by developing new biomarkers and validating innovative molecular diagnostic methods as well as the standardization of sampling collection. As with any laboratory diagnostics, obtaining optimal tissue samples is crucial for achieving reliable and robust results. For the DECODE-VRL study, PPV was therefore selected as the primary sampling method due to its ability to provide high-quality specimens and comprehensive diagnostic information. This first report seeks to optimize the vitreous sampling technique through PPV to facilitate efficient and dependable laboratory diagnostics.

As data are scarce on the effect of gauge sizes on the diagnostic sampling in VRL and there are no data available on direct comparisons, this study aimed to optimize the surgical protocol (20G to 27G – different cutting rates). We evaluated the impact of vitrectomy on lymphocyte integrity using both healthy human peripheral blood lymphocytes and cultured *MYD88*-mutated lymphoma cells. Standardized cell suspensions (1 × 10³, 1 × 10⁴, and 1 × 10⁶ cells/mL) underwent vitrectomy under controlled conditions, with post-procedure viability assessed via flow cytometry. The primary endpoint was viable cell survival across four vitrectomy sizes (20G, 23G, 25G, and 27G). Secondary endpoints included the effects of aspiration alone versus two cutting rates (1500 and 5000 cuts/second), and the influence of initial cell concentration on outcomes.

The findings have immediate clinical relevance, potentially improving the approach to diagnostic vitrectomy in suspected VRL cases and will influence the DECODE-VRL study protocol.

The aim of the DECODE project is to compare a new molecular diagnostic method with the existing molecular diagnostic method for VRL and to test it with a clinical follow-up of 2 years. The aim is to implement a standardized and quality-assured molecular genetic analysis of VRL in specialized centers throughout Germany and thus achieve an improved and more time-efficient diagnosis of this rare and aggressive disease.

By optimizing cell recovery and preservation, this study aims to enhance diagnostic accuracy, reduce the need for repeated procedures, and ultimately improve patient outcomes. The results will offer valuable guidance for vitreoretinal surgeons how the cutting rate and cutter diameter affect cellular integrity and biopsy outcomes in suspected vitreoretinal lymphoma cases and possibly other intraocular malignancies.

## Methods

This study was approved and registered (approval number: 360/2024BO2) by the Ethics Committee of the University of Tübingen. All procedures were conducted in accordance with the Declaration of Helsinki, German legislation, and the guidelines set forth by the local ethics committee.

### Sample Collection and Lymphocyte Isolation

**Figure 1.**
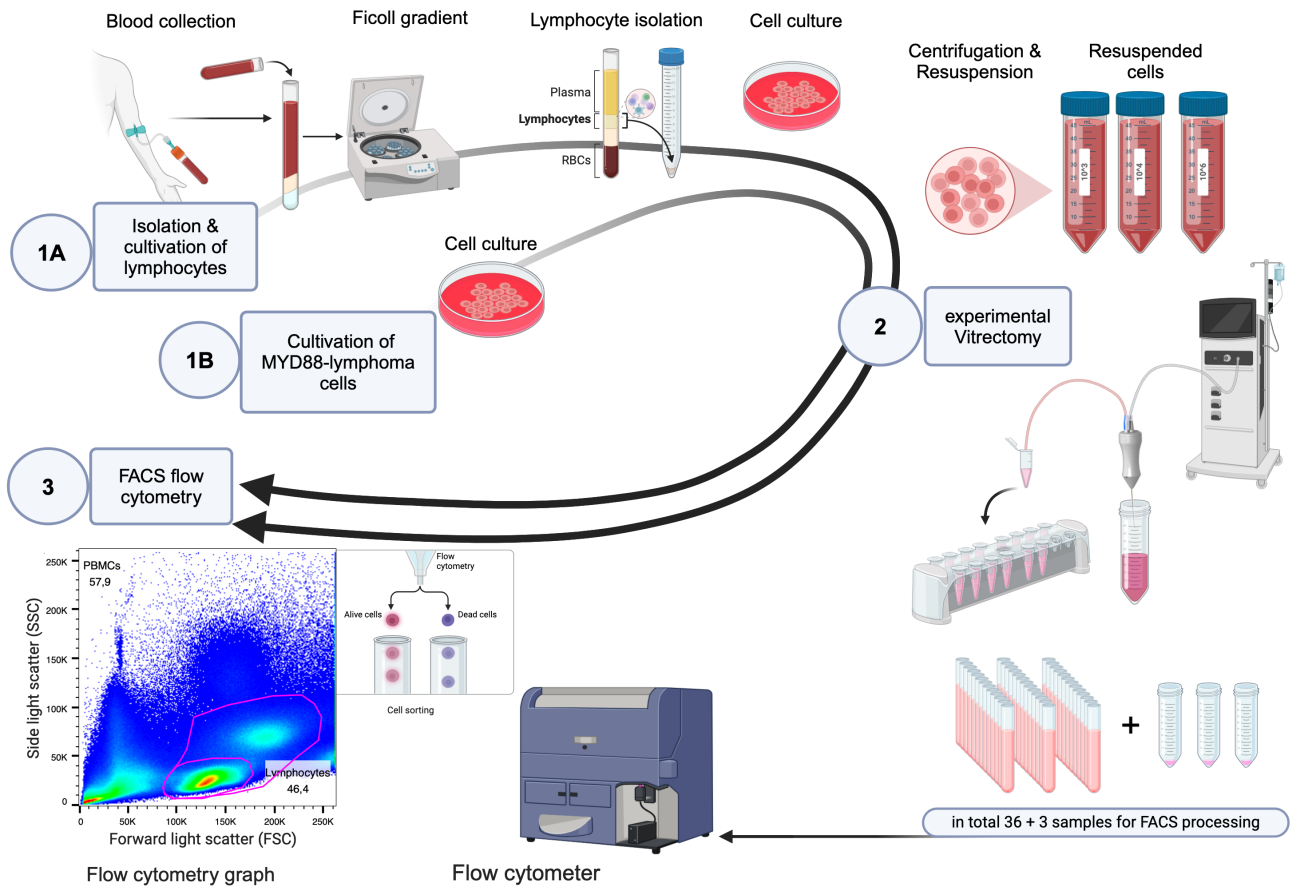
Experimental setting in three steps, (**1A**) for healthy lymphocytes and (**1B**) for diseased lymphoma cells.

Blood (100 mL) from a healthy donor was collected in 9 mL EDTA monovettes (Sarstedt, Germany; Fig. 1^2^). Lymphocytes were isolated via Ficoll gradient sedimentation. The Ficoll-Paque medium (Sigma-Aldrich, USA) and blood sample were centrifuged at 400 G for 30 to 40 minutes at 18°C to 20°C, followed by repeated 0.9% NaCl solution washes. After overnight cultivation (at 37°C in an atmosphere of 5% CO2 and 100% humidity) in lymphocyte separation medium (LSM; Sigma-Aldrich, USA), B lymphocytes in suspension were harvested for the experiment, whereas T lymphocytes settled at the bottom.

**Figure 2.**
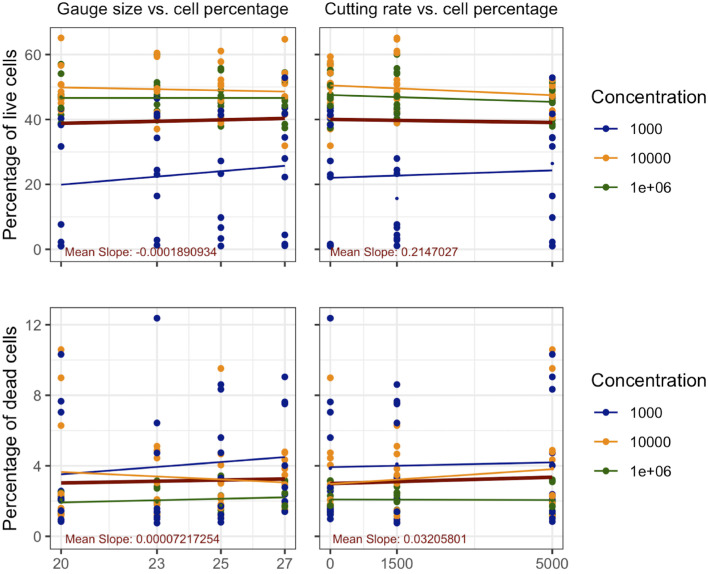
Scatter plot of overall cell survival in respect to gauge size and cutting rate in lymphocytes.

### Cultivation of DLBCL Lymphoma Cells

The human DLBCL cell line (Cellosaurus ID: CVCL_A442) was used for lymphoma cell experiments. Cells were cultivated in RPMI-Medium (Gibco, UK) at 37 degrees and 5% CO2. Splitting was performed every 3 to 5 days.

### Preparation of Test Suspensions and Experimental Vitrectomies

The lymphocyte and lymphoma cell suspensions were aliquoted and diluted into three different concentrations (1 × 10^3^ cells/mL, 1 × 10^4^ cells/mL, and 1 × 10^6^ cells/mL). The volume of each suspension was adjusted with RPMI 1640 Medium (Gibco, UK) to a total volume of 45 mL. Each suspension was filled into a 50 mL Falcon conical centrifuge tube.

Standardized experimental vitrectomies were performed using four cutter sizes (see Fig. 1): 20G single-blade (MIDlabs) and 23G, 25G, and 27G bi-blade (D.O.R.C.) instruments, with the Qube pro vitrectomy system (Fritz Ruck Ophthalmologische Systeme GmbH).

Cell suspensions were inverted three times for uniform distribution before vitrectomy. The probe was centered in Falcon tubes, and 3 mL was aspirated using a syringe at preset cutting speeds. The aspiration was mechanically standardized by pulling to the end of a 5 mL syringe. The exerted aspiration results in a maximum negative pressure of −0.45 bar.^19^ However, this pressure cannot be fully attained due to constant fluid flow, but the standardized procedure ensures constant aspiration pressures in all experimental settings. Samples were sealed, labeled, and stored at room temperature. Each experiment was replicated thrice for all cutter sizes and cell concentrations for both groups (healthy lymphocytes and lymphoma cells). A control sample (3 mL) was retained without vitrectomy from the residual volume of each suspension.

### Fluorescence-Activated Cell Sorting 

Samples were analyzed using an LSR Fortessa X20 (BD Biosciences) and FlowJo software V7.6.5 (FlowJo LLC). Samples were stained with APC-conjugated anti-CD19 antibodies and LIVE/DEAD Fixable Dead Cell Stain (Thermo Fisher Scientific). Cells were incubated on ice for 45 minutes, agitated every 15 minutes, and washed twice with fluorescence-activated cell sorting (FACS) buffer. Flow cytometer gating was set to forward scatter (FSC-A) and side scatter (SSC-A). Raw data were exported to Excel (Microsoft, Redmond, WA) for statistical analysis.

### Statistical Analysis

Statistical analyses were conducted using R statistical software (version 4.3.3; R Core Team,^20^ 2023) on macOS Sonoma 14.5.

The normality of data distribution was assessed using the Shapiro-Wilk test and Q-Q plots. For nonparametric data, Kendall’s tau correlation coefficient was calculated to assess relationships between variables, as this method is appropriate for ordinal data (e.g. gauge size and cutting rate). To verify statistically significant differences between groups, the Kruskal-Wallis test was used as a nonparametric alternative to 1-way ANOVA. The threshold of statistical significance was set at α = 0.05 for all analyses.

## Results

**Figure 3.**
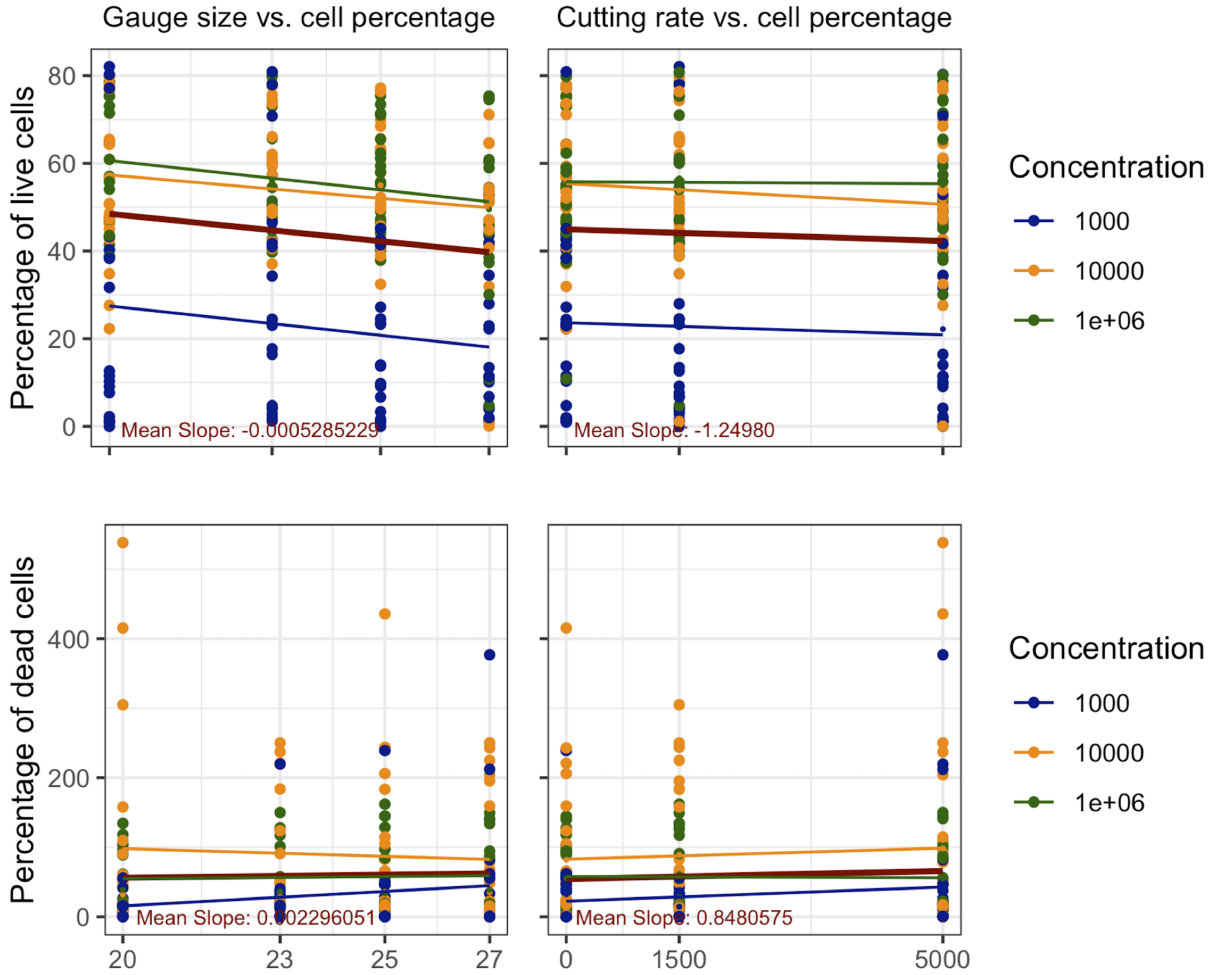
Scatterplot of overall cell survival in gauge sizes and cutting rate in lymphoma cells.

The experiment was conducted three times each for the lymphocytes and lymphoma cells. For each experimental setting, we collected six datapoints for analysis. Thirty-six datapoints could be collected from one experiment, resulting in (see Fig. 2 for lymphocytes and Fig. 3 for lymphoma cells). The Table shows a summary of statistical results for each tested condition.

**Table.** Statistical Analysis of Gauge Size and Cutting Rate Effects on Lymphocyte and MYD88-Positive Lymphoma Cell Viability

Cell Type	Concentration, Cells/mL	Parameter	Kendall's τ (*P* Value)	Kruskal-Wallis χ² (*P* Value)
Healthy lymphocytes	10³	Gauge size	0.1229 (0.3373)	1.4224 (0.7003)
		Cutting rate	0.0230 (0.8618)	1.7432 (0.4183)
	10⁴	Gauge size	0.0289 (0.8214)	0.3694 (0.9465)
		Cutting rate	−0.1150 (0.3841)	3.3168 (0.1904)
	10⁶	Gauge size	−0.0108 (0.9325)	0.2733 (0.965)
		Cutting rate	−0.0843 (0.5233)	0.5466 (0.7609)
	Overall	Gauge size	0.0243 (0.7377)	0.123 (0.9889)
		Cutting rate	−0.0228 (0.7606)	0.1789 (0.9144)
*MYD88-*positive lymphoma cells	10³	Gauge size	−0.1646 (0.1991)	1.8155 (0.6116)
		Cutting rate	−0.2014 (0.1277)	2.4354 (0.2959)
	10⁴	Gauge size	−0.2602 (0.0422)	5.967 (0.1132)
		Cutting rate	−0.0805 (0.5423)	0.5511 (0.7592)
	10⁶	Gauge size	−0.2731 (0.0331)	5.6849 (0.128)
		Cutting rate	0.0096 (0.9422)	1.3429 (0.511)
	Overall	Gauge size	−0.253 (0.00048)	13.337 (0.00396)
		Cutting rate	−0.0485 (0.5165)	0.4505 (0.7983)

The table summarizes the correlations between gauge size and cutting rate effects on MYD88-positive lymphoma cell and healthy lymphocyte viability at different cell concentrations. Notably, a moderate negative correlation between gauge size and cell viability was observed at higher concentrations (10⁴ and 10⁶ cells/mL), with statistically significant Kendall’s τ values.

### Healthy Lymphocytes

Due to a non-normal data distribution of alive lymphocyte count data (in Shapiro-Wilk test and Q-Q plot), combined with the insufficient amount of data (only 3 technical replicates), we were compelled to use nonparametric tests.

#### Correlation of Gauge Size With Cell Viability

**Figure 4.**
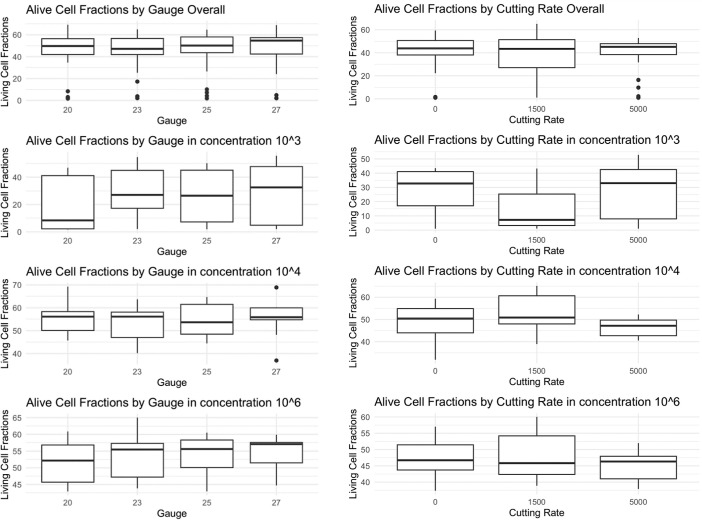
(*Left*) Gauge size and cell survival in lymphocytes. There were no significant correlations for cutting rate and cell survival in different dilutions. (*Right*) Cutting rate and cell survival in lymphocytes. There were no significant correlations for cutting rate and cell survival in different dilutions.

The relationship between gauge size and lymphocyte viability was evaluated at concentrations of 1 × 10³ cells/mL, 1 × 10⁴ cells/mL, and 1 × 10⁶ cells/mL. At 1 × 10³ cells/mL concentration, no significant correlation was found (Kendall’s: τ = 0.1229, *P* = 0.3373; Kruskal-Wallis: χ² = 1.4224, df = 3, *P* = 0.7003). Similar results were observed at 1 × 10⁴ cells/mL concentration (τ = 0.0289, *P* = 0.8214; χ² = 0.3694, df = 3, *P* = 0.9465) and 1 × 10⁶ cells/mL concentration (τ = −0.0108, *P* = 0.9325; χ² = 0.2733, df = 3, *P* = 0.965). These findings consistently demonstrated no statistically significant correlation between gauge size and lymphocyte viability across all concentrations (Fig. 4 *left*).

#### Correlation of Cutting Rate With Cell Viability

The impact of cutting rate on lymphocyte viability was evaluated at concentrations of 1 × 10³ cells/mL, 1 × 10⁴ cells/mL, and 1 × 10⁶ cells/mL. At 1 × 10³ cells/mL concentration, no significant correlation was found (Kendall’s: τ = 0.0230, *P* = 0.8618; Kruskal-Wallis: χ² = 1.7432, df = 2, *P* = 0.4183). Similar results were observed at 1 × 10⁴ cells/mL concentration (τ = −0.1150, *P* = 0.3841; χ² = 3.3168, df = 2, *P* = 0.1904) and 1 × 10⁶ cells/mL concentration (τ = −0.0843, *P* = 0.5233; χ² = 0.5466, df = 2, *P* = 0.7609). These findings consistently demonstrated no statistically significant correlation between cutting rate and lymphocyte viability across all concentrations (Fig. 4 *right*).^21^

### *MYD88*-Mutated Lymphoma Cells

Due to the non-normal distribution of alive *MYD88*-mutated lymphoma cell count data (assessed by Shapiro-Wilk test and Q-Q plot analysis), combined with the limited sample size (3 technical replicates), nonparametric statistical tests were used.

#### Correlation of Gauge Size With Cell Viability

Gauge size effects on lymphoma cell viability were assessed at concentrations of 1 × 10³ cells/mL, 1 × 10⁴ cells/mL, and 1 × 10⁶ cells/mL. At 1 × 10³ cells/mL concentration, non-normal distribution was observed (Shapiro-Wilk: W = 0.71125, *P* = 4.269e-07), with no significant correlation to gauge size (Kendall’s τ = −0.1646, *P* = 0.1991; Kruskal-Wallis: χ² = 1.8155, df = 3, *P* = 0.6116). At 1 × 10⁴ cells/mL concentration, a moderate negative correlation was found (τ = −0.2602, *P* = 0.0422), although Kruskal-Wallis results were inconclusive (χ² = 5.967, df = 3, *P* = 0.1132). The 1 × 10⁶ cells/mL concentration showed a moderate negative correlation (τ = −0.2731, *P* = 0.0331), however, with a not significant difference between groups (χ² = 5.6849, df = 3, *P* = 0.128).

**Figure 5.**
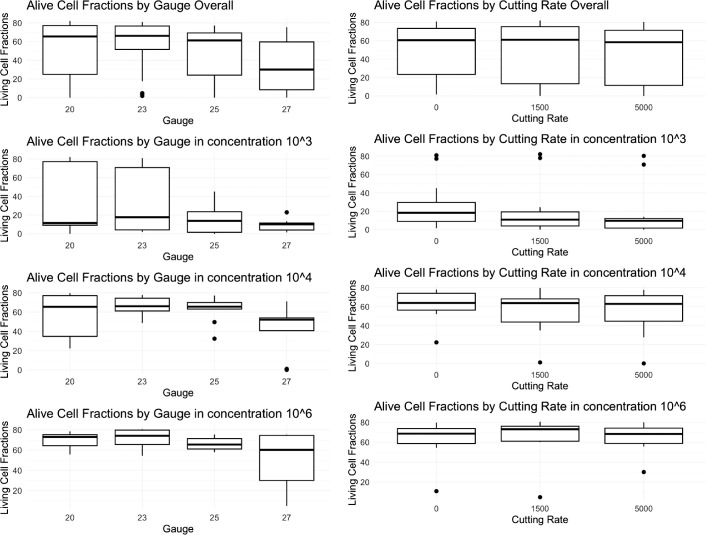
(*Left*) Gauge size and cell survival in lymphoma cells. A correlation of viable cell counts to bigger gauge diameters can be assumed. In 27G cell survival was significantly lower. (*Right*) Cutting rates and cell survival in lymphoma cells. There was no significant correlation for cutting rate and cell survival in different dilutions.

An overall moderate negative correlation between vitrectomy size and cell survival was observed for all lymphoma cell concentrations taken together (Kendall's tau: τ = −0.253, *P* = 0.00048, Kruskal-Wallis: χ² = 13.337, *P* = 0.00396) (Fig. 5 *left*).

#### Correlation of Cutting Rate With Cell Viability

Lymphoma cell viability was evaluated at concentrations of 1 × 10³ cells/mL, 1 × 10⁴ cells/mL, and 1 × 10⁶ cells/mL in relation to cutting rate. At 1 × 10³ cells/mL concentration no significant correlation to cutting rate (Kendall’s: τ = −0.2014, *P* = 0.1277; Kruskal-Wallis: χ² = 2.4354, df = 2, *P* = 0.2959) was observed. Similar results were found at 1 × 10⁴ cells/mL concentration (τ = −0.0805, *P* = 0.5423; χ² = 0.5511, df = 2, *P* = 0.7592) and 1 × 10⁶ cells/mL concentration (τ = 0.0096, *P* = 0.9422; χ² = 1.3429, df = 2, *P* = 0.511). These findings consistently demonstrated no significant correlation between cutting rate and lymphoma cell viability (Fig. 5 *right*).

## Discussion

Diagnosing VRL poses a significant challenge for clinicians. This study aimed to address this issue by optimizing vitrectomy settings to improve vitreous probing. The impact of cutting rate and gauge size was evaluated using two cell-type models: healthy peripheral blood lymphocytes and malignant *MYD88*-mutated lymphoma cells.

There was no evident difference in cell survival for the different gauge size in different dilutions of healthy lymphocytes. Furthermore, the cutting rate also had no measurable effect on cell survival.

However, in lymphoma cells, there was a significantly improved overall cell survival with larger gauge lumina. Different cutting rates, again, had no effect on cell survival.

Our results indicate malignant cells have different biological properties than healthy cells. Whereas shear forces induced by smaller gauge cutters may not affect healthy cells, altered lymphoma cells are more sensitive. Although there are no exact studies on the cell types in question, older studies have indicated that the fragility of leukemia and malignant lymphoma cells is significantly higher than that to peripheral blood lymphocytes.^22^^,^^23^ Cell integrity depends on the complex interplay of protein folding and degradation machineries.^24^ Our findings suggest that these mechanisms may be altered in vitreoretinal lymphoma cells, and that larger cutter sizes are appropriate to preserve these cells for analysis. Further research is necessary to understand the underlying mechanisms of our observations.

When deciding on the right lumen diameter, there is always a trade-off between the durability and flexibility of the instruments, pace of vitreous removal, and wound management. The larger the gauge diameter the larger amount of vitreous a cutter can remove per time. But a greater diameter results in risking an expanded wound leakage, hypotony, and infection.^25^ In summary, this experiment demonstrated that using smaller gauge diameters results in a decreased ease of performance in vitrectomy: the resistance in the sampling tube is higher and the sampling process took longer compared to using conventional 20G or 23G vitrectomy instruments. This is in line with results of several studies published mainly on newer 27G techniques.^25^

Kanavi et al. documented in a case series the cytology results of diagnostic vitrectomies in suspected cases of PVR-LBCL with 25G and contrasted with those reported in the literature for 20G instruments. They found a higher yield with 25G vitrectomies The most recent study included in the analysis on VRL diagnostic yield in 20G was from 2008 and presented data from a 15-year review (1990–2005) of cytopathological findings for VRL.^26^ The superior results for the 25G approach might also be linked to improved vitrectomy settings in general.

Modern systems allow for cutting rates of up to 20,000 cuts per minute (cpm). In this study, the established settings with 5000 cpm were already quite high and probably higher than those normally used in vitreous sampling.

This study used bi-blade or two-dimensional cutting (TDC) vitrectomies except for the 20G cutter. These double-sided blades cut in both the forward and backward directions allowing for higher efficiency. Thus, the duty cycle – defined as the proportion of a cutting cycle in which the vitrectomy port is open – is nearly 100%. Higher duty cycles enable constant aspiration, regardless of cutting speed, thereby reducing turbulences and the time required for a core vitrectomy.^25^^,^^27^^,^^28^

Tekumalla et al. investigated 25G vitrectomy with cutting rates of 500, 1000, 4000, 7500, and 15,000 cpm and found that the cutting rate did not affect lymphoma cell viability. They even used rates up to 15,000 cpm. These findings are consistent with those of our study. In contrast, an older study with 20G recommended only cutting rates up to 1000 cpm for vitreous biopsy in lymphoma suspects.^21^ This resulted in the long-standing recommendation to avoid cutting rates above 600 cpm in diagnostic vitrectomy because they decrease cell viability. However, these results could not be confirmed thereafter. The results are likely linked to older cutter designs and less stable flow dynamics in older machines. The cutter's design and duty cycle may have influenced the results; however, this aspect was not investigated in our study.

Almeida et al. established a prospective laboratory in vitro experiment for bacterial endophthalmitis and diagnostic vitrectomy.^7^ The study was performed with 20-, 23-, and 25-gauge vitrectors at a cutting rate of 0, 1500, and 5000 cpm. This protocol was adopted and expanded by using 27G cutters for our experiment. Viable bacterial colonies were established for all gauges cutting speeds and aspiration rates, with no any statistical difference observed.

Our study improves upon previous works by Jiang et al. and Tekumalla et al., who relied on conventional hemacytometer counting.^16^^,^^29^ We use FACS, which offers rapid, multiparametric analysis of thousands of cells per second. FACS eliminates operator subjectivity and enables cell sorting based on specific characteristics.^30^ Although FACS may be less accurate than counting chambers for routine cell density measurements, its high-throughput capabilities and ability to analyze multiple cellular parameters simultaneously make it superior for comprehensive cell analysis and characterization in this context.^31^^,^^32^

The biphasic nature of the vitreous, which comprises liquid and solid components, leads to distinct biomechanical forces during vitrectomy.^33^ Liquid-phase aspiration follows Hagen-Poiseuille dynamics, wherein smaller gauges (e.g. 27G vs. 23G) exponentially increase flow resistance (∝1/r⁴), thereby influencing sampling efficiency. For solid fragments, Newtonian displacement forces (F = ma) govern particle size. Higher cutting rates reduce deformation and improve fragment aspiration. Lower duty cycles further minimize fragment size.^33^^–^^35^ These principles underscore the need for systematic investigations into optimizing diagnostic yield in VRL through gauge selection and cutting parameters. The influence of these properties on the vitreous needs further investigation.

Our study examined how vitrectomy techniques affect cell counts in a controlled, artificial environment. Whereas these findings suggest potential relationships, limitations include artificially elevated cell densities, a lack of in vivo vitreous properties, and a rather large gained volume. It is important to note that clinical VRL samples likely contain fewer malignant cells, and that other VRL cell lines might react differently depending on cell size and integrity. Therefore, future in vivo studies with larger sample sizes and other cell lines are necessary to confirm these results and assess their relevance to patient care.

A deeper understanding of vitrectomy settings is important for the advancement and optimization of vitrectomy techniques and diagnostic accuracy. Therefore, future studies should aim to identify and differentiate specific factors that significantly influence cell counts during vitrectomy, thereby providing practical guidance to clinicians, enabling evidence-based decision making, and ultimately improving the care of patients with lymphoma. Based on the gained results here, the Decode-VRL study protocol will incorporate the use of 23G and 25G cutters.
